# Nitrogen regulation of transpiration controls mass-flow acquisition of nutrients

**DOI:** 10.1093/jxb/ert367

**Published:** 2013-11-14

**Authors:** Ignatious Matimati, G. Anthony Verboom, Michael D. Cramer

**Affiliations:** Department of Biological Sciences, University of Cape Town, Private Bag X1, Rondebosch 7701, South Africa

**Keywords:** Interception, phosphate, potassium, urea, water flux, water use efficiency.

## Abstract

Transpiration may enhance mass-flow of nutrients to roots, especially in low-nutrient soils or where the root system is not extensively developed. Previous work suggested that nitrogen (N) may regulate mass-flow of nutrients. Experiments were conducted to determine whether N regulates water fluxes, and whether this regulation has a functional role in controlling the mass-flow of nutrients to roots. *Phaseolus vulgaris* were grown in troughs designed to create an N availability gradient by restricting roots from intercepting a slow-release N source, which was placed at one of six distances behind a 25 μm mesh from which nutrients could move by diffusion or mass-flow (termed ‘mass-flow’ treatment). Control plants had the N source supplied directly to their root zone so that N was available through interception, mass-flow, and diffusion (termed ‘interception’ treatment). ‘Mass-flow’ plants closest to the N source exhibited 2.9-fold higher transpiration (*E*), 2.6-fold higher stomatal conductance (*g*_s_), 1.2-fold higher intercellular [CO_2_] (*C*_i_), and 3.4-fold lower water use efficiency than ‘interception’ plants, despite comparable values of photosynthetic rate (*A*). *E*, *g*_s_, and *C*_i_ first increased and then decreased with increasing distance from the N source to values even lower than those of ‘interception’ plants. ‘Mass-flow’ plants accumulated phosphorus and potassium, and had maximum concentrations at 10mm from the N source. Overall, N availability regulated transpiration-driven mass-flow of nutrients from substrate zones that were inaccessible to roots. Thus when water is available, mass-flow may partially substitute for root density in providing access to nutrients without incurring the costs of root extension, although the efficacy of mass-flow also depends on soil nutrient retention and hydraulic properties.

## Abstract (precis)

To test whether N regulates transpiration, *Phaseolus vulgaris* was grown with N placed at one of six distances behind a root-impenetrable mesh whilst control plants intercepted the N-source. N-availability regulated transpiration-driven mass-flow of nutrients from soil zones that were inaccessible

## Introduction

Terrestrial plants transpire 32 trillion tonnes of water vapour annually (Hetherington and Woodward, 2003). Although this is commonly viewed as a by-product of photosynthetic CO_2_ uptake (e.g. Cowan and Troughton, 1971; Monteith, 1988; Kramer and Boyer, 1995), large variations in the rate at which water is traded for each CO_2_ mole assimilated [i.e. water use efficiency (WUE); Hack *et al.*, 2006] as well as evidence for substantial night-time transpiration in photosynthetically inactive C_3_ and C_4_ plants (Caird *et al.*, 2007; Kupper *et al.*, 2012) suggest that transpiration plays an important functional role in plants. Apart from facilitating leaf cooling (Parkhurst and Loucks, 1972; Nobel, 1999) and root to shoot solute transport (Tanner and Beevers, 1990, 2001), transpiration also powers the movement of water and dissolved nutrients to root surfaces by mass-flow (Barber, 1995; Tinker and Nye, 2000; Cramer *et al.*, 2008; Cramer and Hawkins, 2009), reducing rhizosphere nutrient depletion resulting from active nutrient uptake (Scholz *et al.*, 2007; Kupper *et al.*, 2012).

Although mass-flow plays no direct role in uptake across the plasma membrane, the increased rhizosphere solute concentrations may enhance membrane nutrient transport (Cernusak *et al.*, 2011). Thus, transpirational water fluxes appear to play a fundamental role in nutrient acquisition, explaining their up-regulation in plants grown in low-nutrient soils (Wilkinson *et al.*, 2007; Cramer *et al.*, 2008; Kupper *et al.*, 2012) and their down-regulation in plants grown under supraoptimal nutrient supply (Wilkinson *et al.*, 2007). High transpirational water fluxes may be especially important in the acquisition of mobile nutrients or in zones where roots are sparsely distributed (Scholz *et al.*, 2007; Cramer *et al.*, 2008; Kupper *et al.*, 2012). While mathematical models have been used to estimate the spatial extent of nutrient depletion around the rhizosphere (e.g. Rengel, 1993; Syring and Claassen, 1995), the magnitude of the distance over which mass-flow is effective remains unknown. Since knowledge of the spatial scale over which mass-flow operates is highly relevant to our understanding of nutrient acquisition from the soil (e.g. the merits of producing smaller, denser versus larger, sparser root networks), there is a clear need for further work.

Although half a century has passed since water fluxes were first suggested to be important for nutrient uptake (Barber, 1962), the role of nutrients in regulating water fluxes remains poorly understood (Raven, 2008). Several authors have suggested a critical signalling role for xylem nitrogen concentration ([N]) in the regulation of water fluxes (Wilkinson *et al.*, 2007; Gloser *et al.*, 2007; Gorska *et al.*, 2008; Cramer *et al.*, 2009), but this idea lacks substantial empirical support (Gorska *et al.*, 2008). Cramer *et al.* (2008) observed elevated water fluxes in *Ehrharta calycina* (Poaceae) in response to restricted nutrient access; their results suggest a key regulatory role for N. Subsequently, Cramer *et al.* (2009) proposed a model of N regulation in which NO_3_^–^ modulates root hydraulic conductance through its control of aquaporins, and foliar nitric oxide (NO) modulates stomatal conductance (*g*_s_), alongside the regulatory effects of pH and phytohormones. The interaction of these processes is expected to generate a biphasic response of *g*_s_ to [N] (Wilkinson *et al.*, 2007) in which decreasing N availability stimulates *g*_s_ until some threshold value is reached, beyond which it once again decreases, probably due to compromised growth. Existing models have emphasized the role of NO_3_^–^ in regulating water fluxes (Wilkinson *et al.*, 2007; Cramer *et al.*, 2008, 2009; Kupper *et al.*, 2012), neglecting the potential regulatory effects of NH_4_^+^. Given the importance of ammoniacal fertilizers (i.e. including urea) in agriculture (Miller and Cramer, 2004), however, understanding the regulatory effects of ammoniacal fertilizers on water flux is important.

Water use efficiency (WUE) has been found to be more strongly positively correlated with the foliar ratio of N to phosphorus (N:P) than with foliar [N] (Cernusak *et al.*, 2011), identifying foliar N:P as a more likely modulator of WUE than foliar [N]. However, Garrish *et al.* (2010) found that plant water fluxes at a given vapour pressure deficit varied as a function of foliar [N]. The internal to ambient CO_2_ mole fractions, *C*_i_ */C*_a_, and δ^13^C also indicated that water fluxes varied as a function of N availability, but not as a function of P availability (Cernusak *et al.*, 2007; Garrish *et al.*, 2010), suggesting that excess N in the cytosol (measured as foliar N:P) may modulate water fluxes.

*Phaseolus vulgaris* was used here to determine whether N, supplied as urea, regulates water fluxes and consequent nutrient delivery to plants and whether such fluxes display biphasic responses or monotonic decline with distance from the N source. The plants were grown in PVC troughs in which a mesh screen prevented their roots from accessing the N source directly, though they had free access to all other nutrients. The N fertilizer was placed at varying distances from the screen, thus creating a gradient of N accessibility, while a control treatment comprised plants having free access to all nutrients including N. It was hypothesized that plants lacking direct access to N should show higher water fluxes than control plants and that water flux should increase with distance from the N source to an optimum value, beyond which it should decline due to N deficiency.

## Materials and methods

### Plant culture

**Fig. 1.**
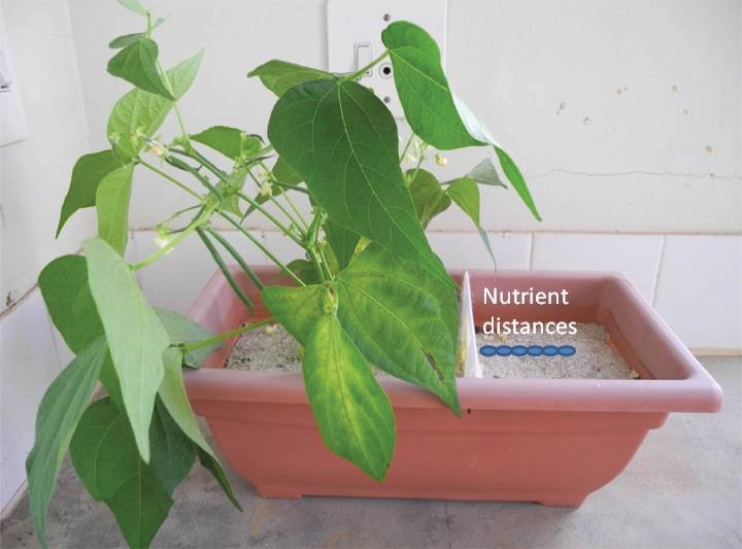
Experimental set-up showing a modified trough containing plants and nutrient placement positions highlighted by circles. A white PVC plate, which has a 25 μm nylon mesh window (30cm^2^), divided the plant and nutrient compartments of the trough. (This figure is available in colour at *JXB* online.)

Forty-two PVC 6 litre troughs (Price and Sons Inc., South Africa) were each divided into two sections, one for growing plants (4 litres) and another for nutrient supply (2 litres), using PVC plates with a 30cm^2^ window covered with a nylon mesh (Nytal 25 μm; Draht-Center, Stuttgart, Germany). The mesh screen restricted roots to one compartment while allowing free transfer of solutes between the compartments (Fig. 1). The plant compartment and the nutrient compartment were filled with 4kg and 2kg, respectively, of thoroughly rinsed acid-washed sand (pH ~7, grade 30/10, Consol Minerals, Cape Town, South Africa). Two *Phaseolus vulgaris* cv. Star 2000 plants (Starke Ayres, Cape Town, South Africa) were planted on one side of the partitioning plate, 5cm from the plate. In the nutrient compartment a 6.5cm^3^ core was excavated in wet sand using a 9mm diameter cork borer at 0, 9, 18, 27, 36, and 45mm from the mesh to allow addition of fertilizers, thus creating a gradient of N availability. The N source was Multicote 4* urea-N (42-0-0 N:P:K, Haifa Group, South Africa), a slow-release granular fertilizer encapsulated in a multilayer polymeric coating. A 5g aliquot of Multicote 4* was placed in the sand cores and they were covered with sand. The use of slow-release urea fertilizer avoids rapid volatilization of N. An independent chemical assay of Multicote 4* indicated a 50% (w/w) [N] and a δ^15^N value of –0.84‰. Because roots could not penetrate the fertilizer compartment, nutrient acquisition was only possible through diffusion and mass-flow of solutes to the roots, and these were consequently termed ‘mass-flow’ plants. Roots of control plants could intercept nutrients, and were consequently referred to as ‘interception’ plants, although both mass-flow and diffusion must also contribute to nutrient mobility in this treatment.

All plants were cultured in a glasshouse at the University of Cape Town for 64 d. Water was supplied sparingly (200ml d^–1^) using spray bottles to prevent leaching of the sand. The sand was maintained at water contents of between 0.15 and 0.2g H_2_O g^–1^ dry weight (DW) sand, which was separately estimated as gravimetric moisture from six additional pots for each treatment. The plant compartment was supplied twice weekly with 200ml of N-free Long Ashton nutrient solution (Hewitt, 1952) containing 2.4mM PO_4_^3–^, 2mM K, 4mM Ca, 1.5mM Mg, 3.5mM SO_4_^2–^, 0.1mM FeEDTA, 0.02mM Mn, 0.14mM H_3_BO_3_, 4.2mM Na, 4mM Cl, 0.003mM Cu, 0.0002mM Mo, and 0.002mM Zn. Plants were exposed to uniform growing conditions by regularly rearranging the positions of the troughs within the glasshouse every second day. The greenhouse received an average light intensity of 1660 μmol m^–2^ s^–1^, and daytime relative humidity inside the greenhouse was ~40% (Power *et al.*, 2010), while the temperatures were kept below 25 °C (day) and above 15 °C (night). After 62 d, the plants were transferred to a growth chamber and left to acclimatize for 48h prior to gas exchange measurements. The growth chambers were equipped with 14×400W HQI-T metal halide lights (Osram Powerstar, Osram, Cape Town, South Africa), 28×400W NAV-T sodium lights (Osram Violox), and 24×150W, 230V incandescent (Sicca, Osram, Cape Town) lamps providing a light intensity of 1000–1200 μmol m^–2^ s^–1^ with 16h light and 8h dark and day/night temperatures of 25 °C/20 °C, with mean day/night relative humidity of ~65%.

### Gas exchange analysis

Gas exchange measurements were performed on the third fully expanded leaf of each plant. All plants were watered before the gas exchange measurements. Photosynthetic rate (*A*), stomatal conductance (*g*_s_), transpiration rate (*E*), and intercellular [CO_2_] (*C*_i_) were determined using a Licor 6400-02B cuvette connected to a portable gas exchange system (LICOR6400, Li-Cor, Inc., Lincoln, NE, USA). Gas exchange characteristics were measured after equilibration in the cuvette (~5min) at a saturating photosynthetically active radiation (PAR) level of 1500 μmol quanta m^−2^ s^−1^ (determined from preliminary light response curves) with 400 μl l^–1^ CO_2_ and a flow rate of 500 μmol s^−1^. Leaf temperature was maintained at 25 °C and relative humidity was ~65% during the measurements. WUE was calculated as *A/E*.

### Biomass measurements

Shoot and root biomass was measured at the end of the 64 d growth period, at their early reproductive stage. There were no apparent differences in the developmental stages of plants, apart from variations in plant sizes. The root systems were carefully excavated onto 2mm^2^ sieves and the sand removed under running water. Above-ground biomass was separated from root material and dried at 70 °C for 48h in a forced draught oven and weighed. Since nodules were absent from all plants, only total root biomass was measured. The shoot biomass of each plant was milled in a Wiley mill using a 0.5mm mesh (Arthur H. Thomas Co., Philadelphia, CA, USA). The milled material was analysed for tissue nutrient concentrations and used for mass spectrometry.

### Foliar elemental and isotope analysis

Foliar nutrient concentrations were determined by ashing the milled leaf material at 480 °C for 8h before dissolving 1:1 (v/v) with HCl (Kalra, 1998). Assessment of the element concentrations in solution was performed using inductively coupled plasma atomic emission spectrometry (Varian Vista MPX, Mulgrave, Australia). To verify the N source used by plants, foliar [N] and ^15^N/^14^N isotope ratios (expressed as δ^15^N) were determined using mass spectrometry. Atmospheric N_2_ fixation is expected to give a δ^15^N signature closest to the natural abundance values of almost zero, whilst urea-N should show an enriched δ^15^N signature due to losses of N through volatilization (Högberg, 1990; Högberg and Johannisson, 1993). Based on variations in foliar δ^15^N, N sources used by plants may be distinguishable (e.g. Vitousek *et al.*, 1989; Robinson, 2001; Cernusak *et al.*, 2009*a*). Between 1.9mg and 2.0mg of ground leaf sample was weighed into a 5 mm×9mm tin capsule (Santis Analytical AG, Teufen, Switzerland). The tin capsules were then combusted in a Thermo Flash EA 1112 series elemental analyser coupled to a Delta Plus XP isotope ratio mass spectrometer via a Thermo Finnigan Conflo III control unit (Thermo Electron Corporation, Milan, Italy). International Atomic Energy Authority standards were used to determine the values.

### Data analysis

One-way analyses of variance (ANOVAs) and post-hoc Tukey’s HSD tests were performed using Statistica (version 10 Statsoft Inc., Tulsa, OK, USA) to evaluate differences in total biomass, shoot:root ratios, water flux, and foliar nutrient content between the fertilizer treatments. Linear models relating total biomass, *E*, *g*_s_, WUE, *C*_i_, *A*, and foliar elemental concentrations to distance from the N source were generated in R (R Development Core Team, 2006). In each instance, model optimality was determined using Akaike information criterion (AIC) scores (Crawley, 2005). Analyses of covariance (ANCOVAs) were used in comparing the slopes of *A* versus *g*_s_.

## Results

### Plant biomass response to N accessibility

**Fig. 2.**
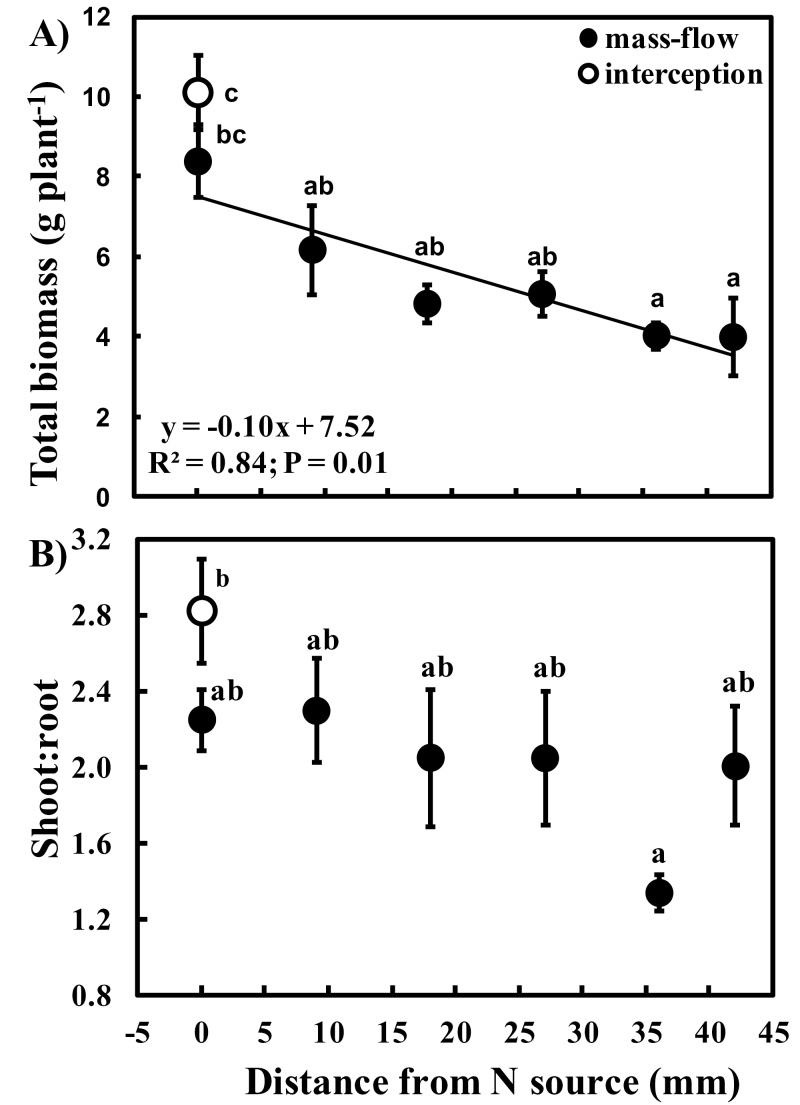
(A) Biomass (g dry weight per plant) and (B) shoot:root ratios of *Phaseolus vulgaris* plants accessing a slow-release urea fertilizer either dibbled around the roots (‘interception’) or placed at six distances from the mesh barrier (‘mass-flow’). Each circle and bar represents a mean ±SE (*n*=6); significantly different means (after a one-way ANOVA with post-hoc Tukey’s HSD, *P* < 0.001 for total biomass and *P* < 0.01 for shoot:root) have different letters. The regression equation relates total biomass to distance from the N source.

The biomass of ‘mass-flow’ *P. vulgaris* plants was negatively correlated with distance from the N source (Fig. 2A), indicating that growth was limited by N availability. When the N source was adjacent to the plants (i.e. plants 0mm from N source), the biomass of ‘mass-flow’ plants was statistically indistinguishable from that of ‘interception’ plants, as were their shoot:root ratios. Despite evidence of increased N limitation with distance from the N source, shoot:root ratios of mass-flow plants did not change significantly with distance from the N source (Fig. 2B).

### Gas exchange response to N accessibility

**Fig. 3.**
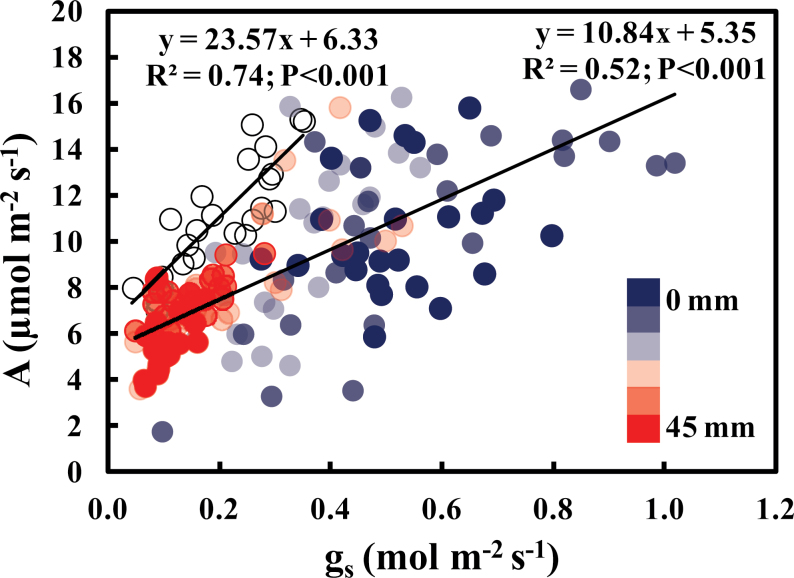
Correlation of stomatal conductance (*g*_s_) with photosynthetic rate (*A*) in *Phaseolus vulgaris* accessing a slow-release urea fertilizer either dibbled around the roots (‘interception’, open circles) or placed at six distances from the mesh barrier (‘mass-flow’, filled circles). Each symbol and bar represents a mean ±SE (*n*=6). The regression equation, coefficient of determination (*R* ^2^), and probability of significance (*P*) are shown on the panel. (This figure is available in colour at *JXB* online.)

Although *g*_s_ and *A* were positively correlated in both ‘mass-flow’ and ‘interception’ plants, the latter showed a steeper slope (Fig. 3), indicating that changes in *g*_s_ and *A* were approximately half as responsive in ‘mass-flow’ as in ‘interception’ plants (ANCOVA interaction term: *t*= –2.495; *P*=0.014). ‘Mass-flow’ plants had a wider range of *g*_s_ values (0.1–1.1 μmol m^–2^ s^–1^) than the ‘interception’ plants (<0.4 μmol m^–2^ s^–1^), suggesting a greater plasticity of response of stomatal conductance in ‘mass-flow’ plants.

**Fig. 4.**
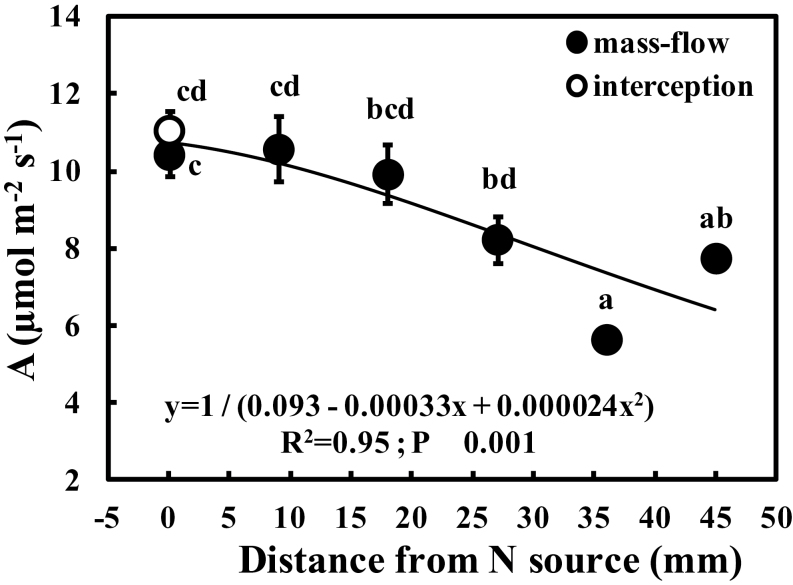
Variation of the photosynthetic rate (*A*) with distance from the N source in *Phaseolus vulgaris* accessing a slow-release fertilizer either by ‘interception’ (open circles) or by ‘mass-flow’ (filled circles). Each circle and bar represents a mean ±SE (*n*=6). Means with different letters showed significant differences after a one-way ANOVA with post-hoc Tukey’s HSD. The regression equation, coefficient of determination (*R* ^2^), and probability of significance (*P*) are shown on the graph.

**Fig. 5.**
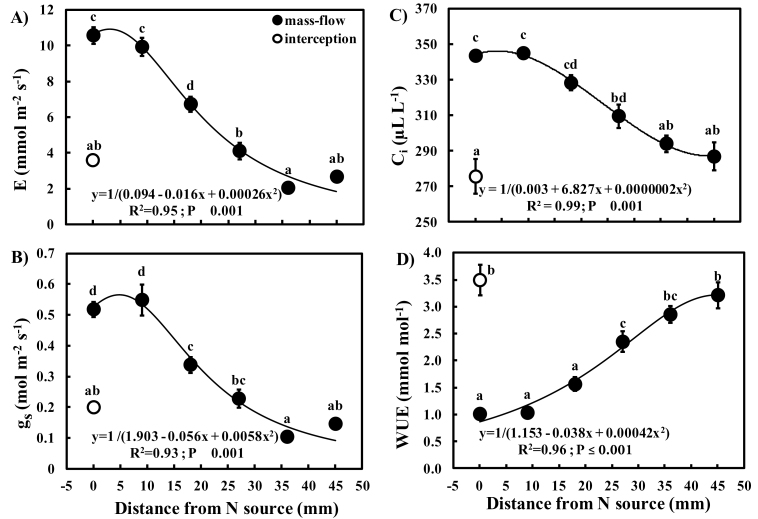
Relationship between distance from N source and (A) transpiration (*E*), (B) stomatal conductance (*g*_s_), (C) internal CO_2_ concentration (*C*_i_), and (D) water use efficiency (WUE) in *Phaseolus vulgaris* accessing a slow-release N-fertilizer either by ‘interception’ (open circles) or by ‘mass-flow’ (filled circles). Each circle and bar represents a mean (*n*=6) ±SE. Means with different letters showed significant differences (at significance value *P*) after a one-way ANOVA with post-hoc Tukey’s HSD. The equations for the fitted lines, coefficients of determination (*R* ^2^), and significance values (*P*) are indicated in each panel.

*A*, *g*_s_, *E*, and *C*_i_ of the ‘mass-flow’ plants all declined with increasing distance from the N source, attaining the highest values over a range of distance from 0mm to 20mm (Figs 4, 5). While ‘mass-flow’ plants adjacent to the N source (0mm) had similar *A* to that of ‘interception’ plants, the former had much higher *E* (2.9-fold), *g*_s_ (2.6-fold), and *C*_i_ (1.2-fold), and lower WUE (3.4-fold). *E* and *g*_s_ of ‘mass-flow’ plants were statistically indistinguishable from those of ‘interception’ plants between 27mm and 45mm from the N source. The changes in *A* and *E* resulted in increased WUE with distance from the N source.

### Foliar nutrient responses to N accessibility

**Fig. 6.**
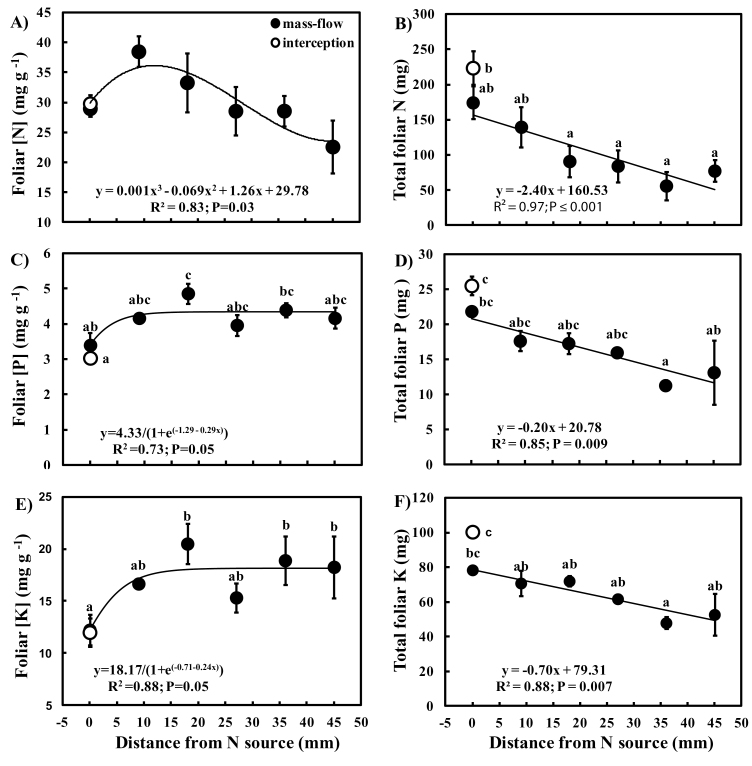
Relationship between distance from the N source and (A) foliar [N], (B) foliar N content, (C) foliar [P], (D) foliar P content, (E) foliar [K], and (F) foliar K content in *Phaseolus vulgaris* accessing a slow-release fertilizer either by ‘interception’ (open circles) or by ‘mass-flow’ (filled circles). Each circle and bar represents a mean (*n*=6) ±SE. Significant differences among distances (one-way ANOVA with post-hoc Tukey’s HSD). The regression equation, coefficient of determination (*R* ^2^), and probability of significance (*P*) are shown on the graph.

Foliar [N] had an asymmetric biphasic relationship with increasing distance from the N source, with a peak at ~10mm (Fig. 6A). Foliar N contents were negatively correlated with distance from the N source (Fig. 6B), corroborating the importance of N in limiting growth. No nodules were observed, and the positive δ^15^N values ranging from 5‰ to 15‰ (mean 11‰, *n*=42), suggest use of a more enriched N source following volatilization of NH_3_ from urea-N (e.g. Högberg, 1990; Högberg and Johannisson, 1993).

**Fig. 7.**
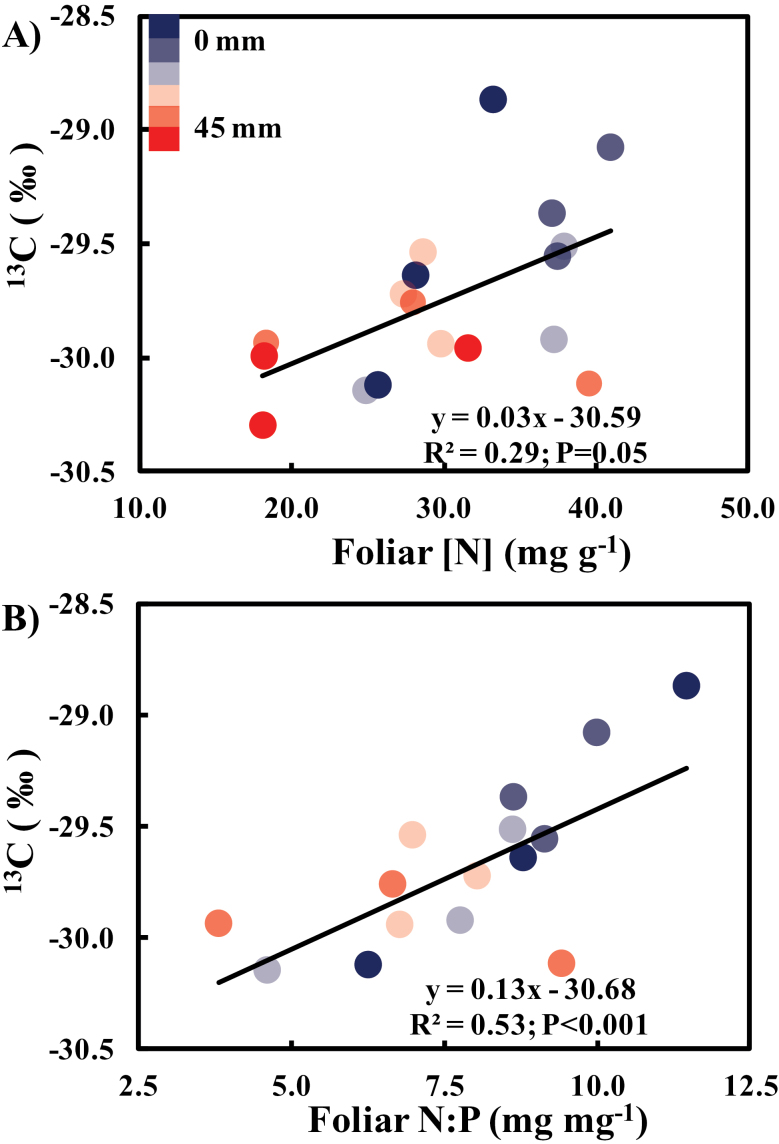
Relationship of (A) foliar [N] and (B) foliar N:P with δ^13^C in *Phaseolus vulgaris* accessing a slow-release fertilizer sequestered from six distances through a mesh-lined substrate compartment. The regression equation, coefficient of determination (*R* ^2^), and the significance (*P < 0.05;**P < 0.01) of the slope are shown on the panel. (This figure is available in colour at *JXB* online.)

Foliar [P] and [K] increased with distance from the N source (Fig. 6C, E), reaching their maxima at peak foliar [N] (~10mm from the N source), and total P and K contents declined as the distance from the N source increased (Fig. 6D, F). To determine whether water fluxes are modulated by foliar [N] or excess N (i.e. N:P), the foliar [N] and N:P ratios were correlated to δ^13^C values (Fig. 7). Plant δ^13^C was used as it provides a time-integrated estimate of intercellular to ambient CO_2_ mole fractions (*C*_i_ */C*_a_) (Farquhar *et al.*, 1982; Brugnoli and Farquhar, 2000), which are a long-term proxy of WUE. δ^13^C was more strongly correlated with N:P (*R* ^2^=0.53; *P* < 0.001) than with [N] (*R* ^2^=0.29; *P*=0.03; Fig. 7). The low δ^13^C values also indicated no water stress in these plants, as may be expected, since the sand was kept close to field capacity.

## Discussion

The present study indicates that N availability partially regulates transpiration, and that transpiration modulates the acquisition of other nutrients. Two lines of evidence support the interpretation that N availability varied with distance from the N source. First, in the absence of nodulation, the supplied N fertilizer constituted the only source of plant-accessible N. The high foliar δ^15^N values indicate that the plants utilized the urea-N from which NH_3_ had been lost through volatilization, resulting in higher δ^15^N values than that of the plants supplied urea (e.g. Högberg, 1990; Högberg and Johannisson, 1993), suggesting that the slow-release fertilizer was the main source of N. Secondly, plant biomass and tissue [N] declined with distance from the N source, indicating N limitations. Although the [NH_4_^+^] derived from the supplied urea was probably higher than that of [NO_3_^–^] in the sand, both NH_4_^+^ and NO_3_^–^ were likely to be present in the sand as a result of hydrolysis and nitrification of urea-N (Sahrawat, 1980). Thus it is not possible to differentiate the effects of the different N forms from these data. The plants were not limited by water and, therefore, variation in transpiration was not a consequence of differences in the available water. This lack of water limitation may facilitate regulation of transpiration by N, resulting in greater water flux when N availability is restricted, but not deficient. Thus, the notion of transpiration as a passive wicking of water from soils by vascular plants when stomata open for CO_2_ uptake (e.g. Nobel, 1999) is questionable and needs to incorporate the regulatory role played by N.

To verify that N regulates transpiration, a common agricultural N fertilizer (urea) was placed at varying distances from the plants, as opposed to varying access to a complex fertilizer containing a suite of nutrients, such as used by Cramer *et al.* (2008). The hypothesis that N regulates transpiration was supported by the higher *E*, *g*_s_, and *C*_i_ values and lower WUE in ‘mass-flow’ than ‘interception’ plants at 0mm from the N source. This was associated with differential responsiveness of *A* to *g*_s_ in ‘interception’ versus ‘mass-flow’ plants. Consistent with changes in WUE, the slope of *A*/*g*_s_ for ‘mass-flow’ plants was half that of ‘interception’ plants and there was a greater range of *g*_s_ in ‘mass-flow’ plants challenged with limited N availability. This indicates that *A* was not strongly limited by *g*_s_, as was also apparent from the higher *C*_i_ values in the ‘mass-flow’ plants <36mm from the N source than in the ‘interception’ plants. Instead, *A* is likely to be limited by demand for photosynthate that is determined by growth rates (e.g. McCormick *et al*., 2008). Nutritionally induced elevation of *E* and *g*_s_ is consistent with a role for transpiration in increasing water flow through soil, thereby compensating partly for reduced availability of N in the rhizosphere. Indeed, low WUE has been observed in a wide range of plants grown with limited nutrients (e.g. Raven *et al.*, 2004).

**Fig. 8.**
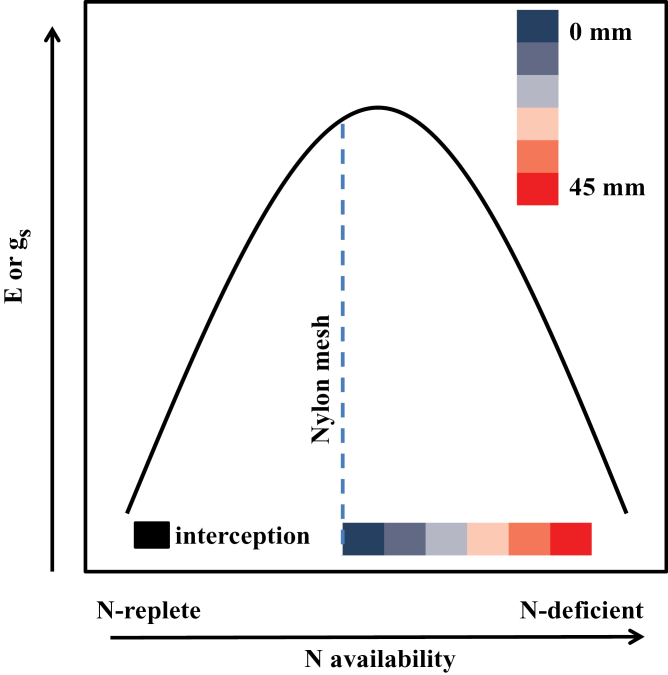
A schematic representation of the effect of decreasing N availability on leaf stomatal conductance (*g*_s_) and transpiration rate (*E*) modified after Wilkinson *et al.* (2007). Excess [N] availability in the ‘interception’ treatment is indicated to result in low *E* and *g*_s_, which increase as N becomes more limiting to compensate for N availability, and then decrease when N is extremely limiting (e.g. 45mm from the N source). The dotted line shows the position of the nylon mesh relative to the N source. (This figure is available in colour at *JXB* online.)

Since ‘interception’ plants had more direct access to the N source, the lower *E* of these plants may be linked to supraoptimal [N], as predicted by Wilkinson *et al.* (2007) (Fig. 8). However, the limitation in access to N imposed on the ‘mass-flow’ plants within 20mm of the N source resulted in strong up-regulation of *E*. The almost monotonic decline in *E* beyond 20mm suggests the down-regulation of *E* evoked by the increasing limitation in N availability, as has been previously observed (e.g. Radin and Parker, 1979; Radin and Ackerson, 1981). The present ‘interception’ and ‘mass-flow’ data seem to match parts of the biphasic trajectory proposed by Wilkinson *et al.* (2007) (Fig. 8). The decline in total biomass with increasing distance from the N source also implies decreasing mass-flow delivery of N. This is consistent with the fact that water flux density at distance (*d*) from the root axis must be proportional to 1/*d* ^2^ (Tinker and Nye, 2000). Despite the gradual N limitation of biomass accumulation with distance from the N source, shoot:root ratios did not vary significantly, suggesting that these plants adjusted their transpiration more than allocation to root biomass for N uptake. Such physiological adjustment of water flux may precede the well-established changes in carbon allocation for adjustment of shoot:root ratios (e.g. Forde, 2002) and accompanying changes in root architecture in response to nutrient supply limitations (Wiersum, 1958; Hackett, 1968; Forde and Lorenzo, 2001).

The higher transpiration rates of ‘mass-flow’ plants were associated with higher foliar [P] and [K], these reaching maximum concentrations when the distance from the N source was 10mm and transpiration was high. Beyond this distance, foliar [P] and [K] remained constant, presumably because of reduced P and K demand as N availability limited biomass production, possibly resulting in feedback suppression of P and K uptake (Marschner and Cakmak, 1986; Valizadeh *et al.*, 2002; Hammond and White, 2008; Tsay *et al.*, 2011). The stronger relationship between δ^13^C and N:P ratios than with foliar [N] may suggest that it is small excesses of N in the cytosol (possibly inorganic N), rather than the overall tissue [N], that is key in the N regulation of transpiration and consequently mass-flow of other nutrients. While accumulation of inorganic N may result in osmotic influences (e.g. Raven, 1985), it is not known whether tissue N represented inorganic or assimilated N. The correlation of N:P ratios with *E* in tropical trees and lianas (Cernusak *et al.*, 2009*b*, 2011) supports the idea that excess N modulates transpiration. Furthermore, despite a significant decreasing trend of foliar [N] with distance, there were no significant differences in foliar [N] (Fig. 6A), possibly indicating that it is N flux that is important in biochemical regulation of *g*_s_ and WUE, as suggested previously (Cramer *et al.*, 2009). The N acquired by roots as NH_4_^+^ is mostly assimilated into amino acids (Miller and Cramer, 2004), and may not alter root hydraulic conductance or the expression of root aquaporins (Guo *et al.*, 2007). Unlike NH_4_^+^, root NO_3_^–^ increases aquaporin-mediated root hydraulic conductivity (Carvajal *et al.*, 1996; Clarkson *et al.*, 2000; Gloser *et al.*, 2007; Gorska *et al.*, 2008) and, when in excess of the capacity of root nitrate reductase, it is taken to the leaves where it is reduced to NO (Cramer *et al.*, 2009) or it can alter xylem sap pH (Mengel *et al.*, 1994; Mühling and Lauchli, 2001), resulting in increased sensitivity of guard cells to abscisic acid (ABA), which elicits stomatal closure (Wilkinson *et al.*, 2004, 2007; Jia and Davies, 2007).

The experimental design used here provided an opportunity to evaluate the spatial scale over which mass-flow is effective in sand. Mass-flow acquisition diminished in effectiveness with distance, with significant reductions in biomass when N was >36mm from the roots. Nevertheless, even at these distances, the plants managed to acquire sufficient N for growth. Whilst sand is experimentally simpler, soils come with a complex cocktail of nutrients and bind different nutrients and nutrient forms to variable extents. This effective mass-flow distance may also vary with soil porosity and texture (Horn *et al.*, 1994), soil moisture (Clarkson, 1981; Gahoonia *et al.*, 1994), and the flux of water to the root. Thus, clay soils with smaller pores, lower hydraulic conductivities (Child and Collis-George, 1950), and greater binding capacity for nutrients may reduce the effective distances for nutrient mass-flow. The limited spatial efficacy of mass-flow and its interactions with soil moisture availability and soil texture are potentially important for understanding the evolutionary ‘choices’ plants make in root system architecture and biomass allocation.

The spatial effectiveness of mass-flow for acquisition of N may have important implications for carbon allocation. The extent to which plants rely on mass-flow may allow a physiological trade-off between the investment in root architecture and the maintenance of water flux. This trade-off may be particularly complex when nutrients and moisture are differently spatially or temporally localized in the soil (López-Bucio *et al.*, 2003; Ho *et al.*, 2005). For instance, in moisture-limited soils, plants would be expected to invest in root biomass for accessing soil nutrients and moisture. In highly permeable soils with abundant moisture, however, ‘mass-flow’ acquisition may complement investment in a costly root system. Whilst mobile elements such as N are known to be acquired through mass-flow (Barber, 1995), acquisition of immobile nutrients such as P are thought to depend largely on root architectural modifications for uptake (e.g. mycorrhizae and cluster roots; Lambers *et al.*, 2006). There is, however, evidence that P acquisition also benefits from mass-flow (Cernusak *et al.*, 2011). This may be particularly the case for more mobile organic P or in soils with low binding capacity for P. Overall, the recognition that N partially regulates transpiration and thus mass-flow of N and possibly other nutrients is important. In a warming global climate where water supplies are dwindling (Wilkinson and Hartung, 2009), strategic N fertilization may provide an opportunity for moderating plant water demands.

## Abbreviations:

A: photosynthetic rate
*C*_a_: atmospheric [CO_2_]
*C*_i_: intercellular [CO_2_]
*E*: transpiration rate
*g*_s_: stomatal conductance
PAR: photosynthetically active radiation
WUE: water use efficiency.
